# Axillary management in early breast cancer with onset surgical management and positive sentinel lymph node

**DOI:** 10.3332/ecancer.2021.1193

**Published:** 2021-03-01

**Authors:** Lia Pamela Rebaza Vasquez, Jaime Ponce de la Torre, Raul Alarco, Joseana Ayala Moreno, Henry Gomez Moreno

**Affiliations:** Unit of Basic and Transnational Research, Oncosalud-AUNA Clinic, Lima, Peru; a https://orcid.org/0000-0002-9620-9460

**Keywords:** breast cancer, positive sentinel lymph node, radical axillary dissection

## Abstract

Over the years, the management of early breast cancer has evolved by leaps and bounds, as has the concept of axillary staging and armpit surgical management. Five randomised studies exist that evaluate the possibility of omitting regional locus surgical axillary treatment in patients with early breast cancer and positive sentinel lymph nodes without it having an impact on the prognosis of the disease in selected cases. A review of the literature on the management of the axilla in early breast cancer is presented.

## Introduction

Over the years, breast cancer management has evolved in leaps and bounds. We have moved from radical procedures in the era of Halsted [1] and Urban [2] to the era of conservation surgery with Veronesi [3] and Fisher [4], followed by the change in the surgical approach of the axilla with sentinel lymph node validation as an axillary staging method and the omission of radical axilla dissection in patients with negative sentinel nodes and even in selected patients with positive sentinel lymph nodes as described by Giuliano [5] in Z0011. Despite this, there is still a lot of controversy with respect to the surgical management of the axilla. There is fear of global de-escalation and ignorance of the real impact that locoregional treatment has on breast cancer prognosis. The objective of this review is to show evidence to date to establish a clearer concept regarding the management of the axilla in early breast cancer with positive sentinel lymph nodes.

## Sentinel lymph node history

The concept of the sentinel lymph node was described by Ramón Cabanas and colleagues in 1977, with their proposed idea being to identify by lymphangiography the first nodes to which the lymphatic flow of the penis is directed, and after removal, carry out microscopic study of the same to determine the need or not to perform complete nodal dissection [6]. Afterwards, Morton published a study using sentinel lymph nodes in melanoma with a technical modification, by using patent blue instead of lymphangiography, with positive results [6]. At the same time, with published data from the National Surgical Adjuvant Breast and Bowel Project (NSABP) 04, the need for radical axillary dissection (RAD) for all patients with early breast cancer began to be questioned [7]. In 1994, Giuliano applied the technique described by Morton for breast cancer, demonstrating that the identification and study of the sentinel lymph node was a reliable marker to detect axillary metastasis [8].

If we analyse the data from NSABP 04 published in 2002, which included 1,079 patients with early N0 breast cancer and randomise the management of radical mastectomy (mastectomy and RAD), total mastectomy without axillary management and total mastectomy with radiotherapy, we will see that 40% of the patients that underwent radical mastectomy had axillary compromise. This is to say that approximately 40% of the patients that did not have axillary surgery were left with nodal disease in both the radiotherapy group and the non-treatment group without significant impact on either overall survival (OS) nor in disease-free time (DFT) after 20 years of follow-ups.

The scientific validation of the sentinel lymph node in breast cancer was given in 2000 and 2003 with studies by Giuliano [9], Giuliano [10] and Veronesi [11], respectively. They demonstrated that the omission of axillary dissection in patients with negative sentinel lymph nodes was oncologically as safe as axillary dissection, but without the same conditional morbidities. Since then, these discoveries have been supported by multicentre studies such as the NSABP-32, in which it was found that OS and disease-free survival of sentinel lymph node biopsy patients versus those that had axillary dissection were similar (hazard ratio (HR): 90.3%; 95% CI: 88.8%–91.8% versus HR: 91.8%; 95% CI: 90.4%–93.3%) (HR: 81.5%; 95% CI: 79.6%–83.4% versus HR: 82.4%; 95% CI: 80.5%–84.4%) [12]. Thus, the sentinel lymph node was consolidated as the elected method for surgical staging of the axilla in patients with clinically negative axillary nodes, reserving the RAD for patients with positive sentinel lymph nodes or clinical evidence of initial axillary compromise.

Since axillary management has changed further, the omission of RAD in positive sentinel lymph node breast cancer or even the use of sentinel lymph nodes after neoadjuvant therapy in breast cancer patients and complete clinical axillary response patients is currently being considered [15].

## Omission of locoregional axillary treatment in early breast cancer patients, initial surgical management and positive sentinel lymph node

To date, five randomised studies exist that evaluate the possibility of omitting locoregional axillary treatment in early breast cancer and positive sentinel lymph node patients. Of the five studies, three (ACOSOG-Z0011, IBCSG 23-01, AATRM) compare the observation (OBS) of the axilla versus RAD, and two (AMAROS, OTOASOR) compare axillary radiotherapy (AR) versus RAD (Table 1).

### Studies that compare axillary surgical management (axillary radical dissection) versus OBS

The ACOSOG study initially published in 2011 is perhaps the most important and controversial study that addresses this topic. It included a total of 856 patients with early breast cancer (T1–2, N0) with up to two positive sentinel lymph nodes (micro and/or macro metastasis) who underwent initial conservation surgery (Table 2). These were randomised into two groups for axillary management: one was RAD (436) and the other was OBS (no RAD) (420). After 10 years of follow-up, in the 2016 publication, it was reported that there was no significant difference between the OBS group versus the RAD group, nor in the OS (83.6%, 86.6% *p* = 0.25), nor in the DFT (80.2%, 78.2% *p* = 0.31), nor in axillary recurrence (1.3%, 0.6% *p* = 0.44) [5].

Another study that considered the omission of axillary treatment in positive sentinel lymph node patients is the IBCSG 23-01. This European study includes 934 patients with early breast cancer (T2, N0) with at least one sentinel lymph node with micrometastasis. The patients were randomised for axillary management in two groups; one was for RAD (464) and the other was for OBS (no RAD) (467). After 10 years of follow-up, there was no significant difference between the group that was under OBS versus the group that underwent RAD, nor in the DFT (76.4%, 74.4% *p* = 0.24), nor in the OS (90.8%, 88.2% *p* = 0.2) [13].

The AATRM 048 also evaluated the same topic. It studied 233 patients with invasive breast cancer with tumours of up to 3.5 cm and clinically negative axillae with positive sentinel lymph nodes for micrometastasis. The patients were randomised for axillary management in two groups; one was for RAD (121) and the other was for OBS (no RAD) (112). After 5 years of follow-up, there was no significant difference between OBS versus RAD in the OS (98.2%, 98.4% *p* = 0.3) [16].

### Studies that compare two types of axillary treatment: surgical axillary management (RAD) versus AR

Studies that compare AR versus axillary surgery (RAD) highlight the AMAROS study. AMAROS includes 4,806 breast cancer patients with T1–T2, N0 and positive sentinel lymph nodes for macrometastasis (Table 2). The patients were randomised into two groups for axillary management; one underwent RAD (744) and the other underwent AR (25 fractions of 2 Gy in the three axillary groups and supraclavicular nodes). After 10 years of follow-up, no significant difference was found, nor in OS (84.6%, 81.4% *p* = 0.26) nor in the DFT (81.7%, 78.2% *p* = 0.19). Despite this, the assessment of non-inferiority had little power given the few events recorded [14].

The OTOASOR study recruited 474 T1, T2, T3 and N0 breast cancer patients that had positive sentinel lymph nodes for macrometastasis. The patients were randomised into two groups; one underwent RAD (244) and the other underwent AR (50 Gy in 25 sessions in the three axillary groups and the supraclavicular fossa) (231), with a median follow-up of 8 years. There was no significant difference between the two groups, either in OS (77%, 84% *p* = 0.06) or DFT (72.1%, 77.4% *p* = 0.6) [17].

**Figure 1.**
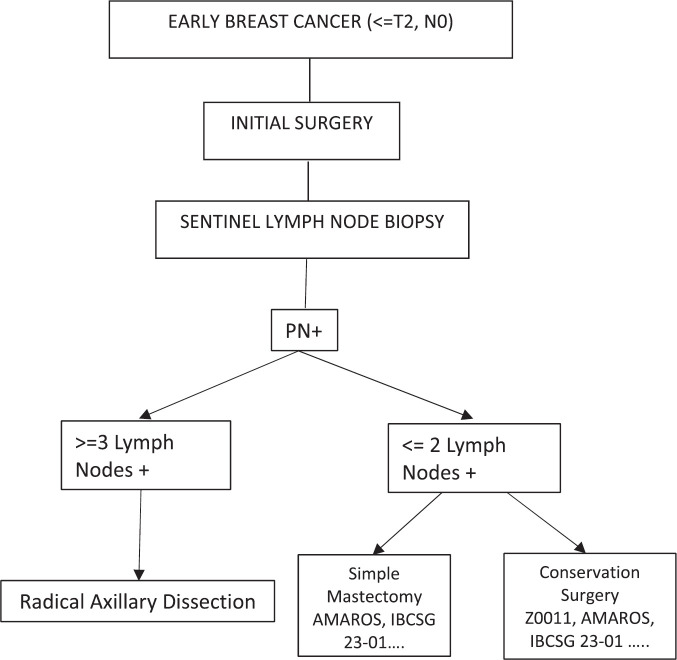
If one has three or more positive sentinel lymph nodes, the indication would be radical axillary dissection, and if one has up to two positive sentinel lymph nodes, they could be chosen for OBS or AR depending on the type of surgery performed on the breast (PN+: positive lymph nodes by pathology).

No significant difference was observed within the described studies between treating or omitting standard axillary treatment, either by opting for OBS or AR. Therefore, we can now conclude that the decision regarding whether or not to perform RAD for early breast cancer patients who go to surgery initially with positive sentinel lymph nodes will depend on the amount of disease found. If one has three or more positive sentinel lymph nodes, the indication would be RAD, and if one has up to two positive sentinel lymph nodes, they could be chosen for OBS or AR depending on the type of surgery performed on the breast (Figure 1).

We are currently in a de-escalation stage in breast cancer treatments, so we should consider making decisions based on the probable benefits according to the disease stage and the tumour biology, always taking into account the intensity and duration of the secondary effects and the impact they will have on the patients’ qualities of life [19]. One of the main problems of axillary treatment in breast cancer is the morbidity of this condition in the superior limb (lymphedema, paresthesia, functional limitation), so de-escalating the treatment with the safety that studies show should be the best option. Despite this, resistance exists in treating physicians to more precise axillary surgical management as reported by Morrow *et al* [20] in 2018: ‘There is a variation in the surgeons’ acceptance of more limited surgical management for breast cancer and their acceptance is conditioned by the number of patients treated and the number of multidisciplinary activities’ [20].

If we analyse why there is so much resistance to the omission of axillary treatment, we will see that one of the main factors is the fear of not treating the disease or axillary compromise locoregionally. If we review the studies carefully, we will see that the percentage of untreated axillary nodal involvement in patients who did complete RAD was between 13% and 38.5%. This is to say that in patients that did not have axillary treatment or had axillary treatment with AR, there was an untreated nodal involvement ranging from 13% to 38% [5, 13, 14, 16, 17]. This questions the treatment of the underarm as seen in breast cancer. Does the locoregional axillary treatment actually impact the prognosis of our patients or should we only use axillary surgery as a form of staging? This is not a new idea. If we analysed the data from NSABP 04 published in 2002, which included 1,079 patients with early N0 breast cancer and randomised the management of radical mastectomy (mastectomy and RAD), total mastectomy without axillary management and total mastectomy with radiotherapy, we will see that 40% of the patients that underwent radical mastectomy had axillary compromise. This is to say that approximately 40% of the patients that did not receive axillary surgery were left with nodal disease in both the radiotherapy group and the non-treatment group without significant impact in OS or DFT after 20 years of follow-up [7]. With all of this information, we can infer that the omission of locoregional axillary treatment would not have an impact on the prognosis of early breast cancer, especially in a selected group of patients.

## Pre-decision axillary ultrasonography: should the sonography change our behaviour?

This is a very controversial question and many are probably at odds or in doubt when discussing it. But we should begin by remembering that, within the studies described above, which validate the omission of RAD in positive sentinel lymph nodes patients, more than 60% did not have a preoperative axillary ultrasound evaluation (Table 3). Further, if we only include the studies that compared surgical axillary treatment versus solely observing (ACOSOG Z0011 and the IBCSG 23-01 study), we will see that none of the patients had axillary ultrasound evaluations prior to surgery.

**Figure 2.**
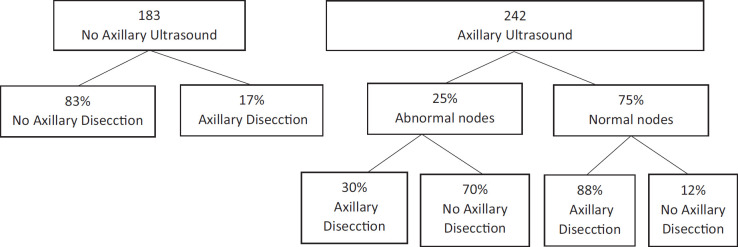
70% of patients with ultrasonographically abnormal or suspected lymph nodes did not require RAD.

Melissa Pilewski [22], in a retrospective analysis, evaluated 425 patients with early breast cancer that underwent conservation surgery, positive sentinel lymph node biopsy and also met the Z0011 criteria. From this, 242 patients had axillary ultrasounds previous to surgery, where abnormal lymph nodes were found in 25% of cases. After analysing those requiring axillary dissection using the Z0011 criteria, of the 183 patients that did not have axillary ultrasounds previously, 17% of the cases required axillary dissection. However when we analyse the patients that had axillary ultrasounds previously, we find that in patients with ultrasonographically normal lymph nodes, only 12% required axillary dissection, and in patients with ultrasonographically altered lymph nodes, only 30% required axillary dissection according to the mentioned criteria. This is to say, 70% of patients with ultrasonographically abnormal or suspected lymph nodes did not require RAD [21] (Figure 2).

If we go further and ask ourselves: Can a positive axillary lymph node biopsy in a patient with early breast cancer and clinically negative axilla predict via an efficient manner the volume of axillary disease and thus define the need for RAD?

**Figure 3.**
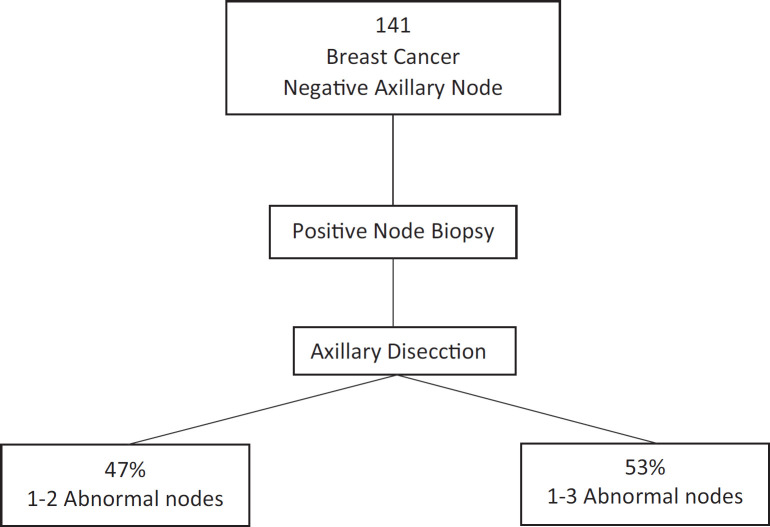
Positive axillary biopsy in patients with clinically negative axillae would lead to unnecessary radical dissection in more than 40% of cases.

Melissa Pilewskie [21] also evaluated this question. They studied 141 women with early breast cancer without clinical axillary compromise that had axillary ultrasounds and positive axillary biopsies for metastasis before surgery. Of all of these, 47% had from one to two positive lymph nodes at the moment of RAD. This is to say that the RAD may have been omitted. Furthermore, they observed that 53% of patients that had more than three positive lymph nodes during RAD had not only positive needle biopsy but also more than one suspected imaging lymph node in the vast majority of cases (*p* = 0.003). Therefore, the authors could conclude that we cannot predict with a positive axillary biopsy the volume of axillary disease in patients with clinically negative axillae with a certain manner. On the contrary, this leads us to perform unnecessary radical dissection in more than 40% of cases (Figure 3) [22].

Currently, the work that reports the applicability of Z0011, for the most part, does not utilise axillary ultrasonography prior to surgery. A clear example is the study published by a group from Memorial Sloan Kettering in 2017 [18], but is this something we should generalise?

While we could question axillary ultrasonography prior to surgery in selected cases, there is still much to analyse with respect to this topic and future work will give us more light. We must evaluate each case individually today.

## It is necessary to irradiate the axilla in all patients in which RAD is omitted and also have positive sentinel lymph nodes

Before we begin to address this issue, let us remember the phrase that Dr Mónica Morrow mentioned in the Sant Gallen of 2017: ‘Bigger surgery does not solve the problem of a bad biology’. Let us analyse this phrase and focus only on surgery. Should we not question locoregional management? Could not we say that greater locoregional management would not resolve the problem of a bad biology? The de-escalation of treatments is a process that has been taking place and presents, as everything changed, some resistance. We will now analyse the evidence for this specific point.

As we explained previously, the Z0011 is one of the pillars that leads to the omission of axillary treatment in early breast cancer patients with conservation surgery and positive sentinel lymph nodes (macrometastases 1–2, without extracapsular extension). Despite this, it is one of the most criticised studies [5].

Reshma Jagsi [23] in 2014 evaluated radiotherapy as a possible cause of the prognosis in Z0011 patients in which they omitted axillary surgical treatment. Only 605 reports of radiotherapy treatment were studied for all Z0011 patients (856). Of these 605 patients, only 540 (89%) of patients received breast radiotherapy, i.e. 11% of evaluated patients did not receive any type of radiotherapy treatment [23]. Of the 540 patients that received radiotherapy, 15% received treatment in the supraclavicular region. However, the only significant factor related to the decision to irradiate or not at the supraclavicular level was the number of positive axillary lymph nodes, with patients with three or more positive lymph nodes being most of whom received irradiation at the supraclavicular level. Another point that Jagsi [23] emphasised is that they could only obtain detailed data of radiotherapy treatment in 29% of cases, i.e. of 228 patients (124: sentinel lymph node biopsy, 104: RAD). Of the 124 patients that did not receive axillary surgical treatment, only 83% received breast radiotherapy with tangential fields (breast only) and only 40 cases received breast radiotherapy with high tangential fields, i.e. axillary fields I and II were included [23].

**Figure 4.**
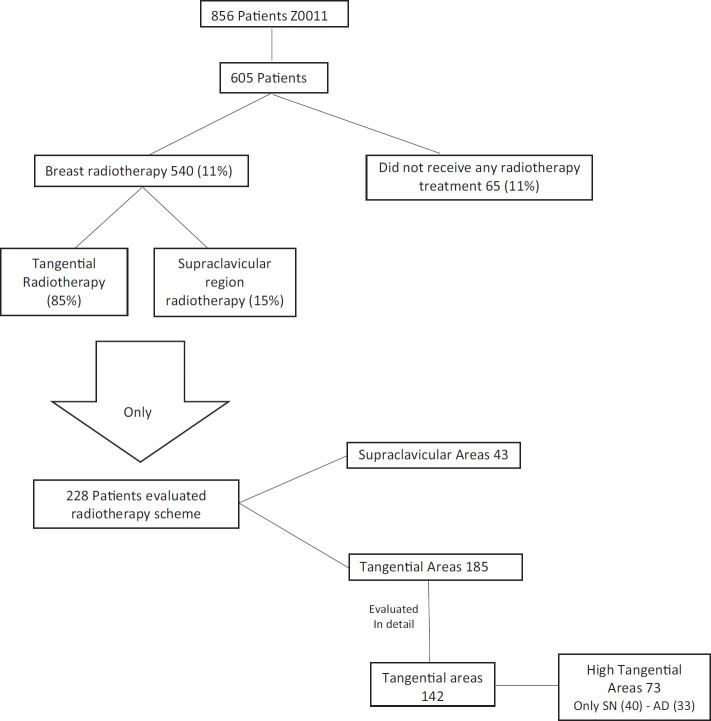
Review of work by Jagsi [23], Radiation Field Design in the ACOSOG Z0011 (Alliance).

Now by cold-analysing the information, it is true that a tangential field to the breast will irradiate axillary groups I and II indirectly, but at a non-treated dose, i.e. at a suboptimal dose. Furthermore, it should be remembered that 11% (66 patients) of the 605 patients did not receive any radiotherapy treatment in the breast nor the axilla and that only 40 cases (6% of the total) of the patients that did not receive RAD receiver radiotherapy treatment at the axillary groups I and II level. Thus, we would have to ask again: Is it necessary to treat the axilla locoregionally with either surgery or radiotherapy in 100% of cases? [23] (Figure 4)

Morrow [18] raised the same question; in 2017, they published a prospective validation study that included 793 patients. All of the patients had conservation surgery and sentinel lymph node biopsies. Only 130 patients underwent radical axillary surgery, with the most frequent cause being the presence of more than three positive sentinel lymph nodes or extracapsular compromise. The overall 5-year survival of the patients who went to sentinel lymph node biopsy was 93% (IC 89%–94%). The irradiation fields were analysed in 509 patients where the information was available, excluding 25 that did not receive standard treatment (partial breast radiotherapy or no radiotherapy treatment). 21% of the patients received prone breast radiotherapy, 58% of the patients received tangential supine breast radiotherapy and 21% received axillary and breast radiotherapy. It should be noted that the patients who had AR had increased risk factors [18]. After 37 months of follow-up, there were five node recurrences, of which four were ganglial and at a distance and one was ganglial and to the breast. The accumulated risk to 5 years of ganglial reoccurrence was 1%, and there was no significant difference depending on the type of radiotherapy received by the patients [18].

If we analyse the findings to date, we can conclude that the results initially presented by Z0011 are valid, so patients with T2, N0 breast cancer undergoing conservation surgery and up to two positive sentinel lymph nodes without extracapsular extension should not receive local axillary treatment (surgery or radiotherapy). Nevertheless, there is still a lot of controversy on this issue, as while it is true that there is a group of patients did not receive axillary and breast radiotherapy, those who received indirect tangential breast radiotherapy may have been ‘partially treated’ although at suboptimal doses. It remains to be defined today whether or not there is a group of patients who benefit from locoregional treatment of the axilla or if it is simply something we should stop doing in this scenario. The POSNOC Trial promises to finish absolving many of our doubts on this and other scenarios. POSNOC is a pragmatic, multicentre, non-inferiority and randomised trial that includes patients with T2 and N0 breast cancer with up to two sentinel lymph nodes with macrometastases that regardless of the type of breast surgery are randomised for axillary management, either OBS or axillary treatment via surgery or radiotherapy [24].

## Conclusions

Based on the current scientific evidence, we consider that in patients with early breast cancer and up to two positive sentinel lymph nodes with extracapsular extension who have initial conservation surgery, axillary surgical treatment can be omitted. Furthermore, given the controversy that still exists, it is recommended to consider the possibility of the omission of AR in an individual manner within a multidisciplinary team.

## Funding statement

The review has been self-funded.

## Conflict of interest

No conflicts of interest exist.

## Figures and Tables

**Table 1.** Randomisation of studies that evaluate the omission of locoregional axillary treatment in patients with early breast cancer and positive sentinel lymph nodes.

Tests	Randomisation	
	OBS versus RAD	RAD versus AR
ACOSOG Z0011 (856) [5]	50% macrometastasis, micrometastasis No extracapsular extension	
IBCSG 23-01 (933) [13]	Micrometastasis	
AATRM (233) [16]	Micrometastasis	
AMAROS (1,425) [14]		Macrometastasis 60% micrometastasis
OTOASOR (474) [17]		Macrometastasis, 68% micrometastasis

RAD, Radical axillary dissection; AR, Axillary radiotherapy

**Table 2.** Summary table of randomised studies on omission of axillary management and positive sentinel lymph nodes.

	ACOSOG Z0011 [5]	IBCSG 23-01 [13]	AATRM [16]	AMAROS [14]	OTOASOR [17]
No. of patients	Total RAD: 436 OBS: 420	Total RAD: 464 OBS: 467	Total RAD: 121 OBS: 112	Total RAD: 744 AR: 681	Total RAD: 244 AR: 231
Median follow-up	9.25 years	9.7 years	5 years	10 years	8 years
(%) Conservation surgery	100%	91%	84%	83%	88%
Positive lymph nodes in RAD	27.3%	13%	13%	33%	38.5%
A.Re. DFT OS	OBS RAD 1.3 0.6 *p* = 0.44 80.2 78.2 *p* = 0.31 83.6 86.6 *p* = 0.25	OBS RAD 2 <1 (8 *p*) 76.4 74.4 *p* = 0.24 90.8 88.2 *p* = 0.2	OBS RAD 1 0 98.2 98.4 *p* = 0.3	AR RAD 1.19 0.43 81.7 78.2 *p* = 0.19 84.6 81.4 *p* = 0.26	AR RAD 2 1.7 *p* = 1 72.1 77.4 *p* = 0.6 77 84 *p* = 0.06

A.Re, Axillary recurrence; RAD, Radical axillary dissection; OBS, Observation; DFT, Disease-free time; OS, Overall survival; AR, Axillary radiotherapy Source: Table 2 translation, Morrow [18]

**Table 3.** Preoperative axillary ultrasound evaluation in studies that evaluate the omission of locoregional axillary treatment in patients with early breast cancer and positive sentinel lymph nodes.

Study	Axillary ultrasound Preoperative	No axillary ultrasound Preoperative	Total
AMAROS [14]	859 (60%)	566 (40%)	1,425 (100%)
IBCSG 23–01 [13]	0	931 (100%)	931 (100%)
ATTM [16]	3 (1.2%)	230 (98.8%)	233 (100%)
OTOASOR [17]	474 (100%)	0	474 (100%)
ACOSOG-Z0011 [5]	0	856 (100%)	856 (100%)
	1,336 (35%)	2,583 (65%)	3,919 (100%)
